# Negative Thermal Expansion in Ba_0.5_Sr_0.5_Zn_2_SiGeO_7_

**DOI:** 10.3390/ma9080631

**Published:** 2016-07-27

**Authors:** Christian Thieme, Christian Rüssel

**Affiliations:** Otto-Schott-Institut für Materialforschung, Jena University, Fraunhoferstr. 6, Jena 07743, Germany; ccr@uni-jena.de

**Keywords:** X-ray diffraction, negative thermal expansion, phase transition

## Abstract

Solid solutions with the composition Ba_0.5_Sr_0.5_Zn_2_Si_2-x_Ge_x_O_7_ and BaZn_2_Si_2-x_Ge_x_O_7_ were prepared with different values of x using a conventional mixed oxide route. Both compounds exhibit very different thermal expansion, which is due to the different crystal structures. Ba_0.5_Sr_0.5_Zn_2_Si_2-x_Ge_x_O_7_ solid solutions exhibit the structure of high-temperature BaZn_2_Si_2_O_7_ and show negative thermal expansion, which was proven via high-temperature X-ray diffraction. Up to around x = 1, the crystal structure remains the same. Above this value, the low-temperature phase becomes stable. The Sr-free solid solutions have the crystal structure of low-temperature BaZn_2_Si_2_O_7_ and show also a limited solubility of Ge. These Sr-free compositions show transitions of low- to high-temperature phases, which are shifted to higher temperatures with increasing Ge-concentration.

## 1. Introduction

The phase BaZn_2_Si_2_O_7_ exhibits a phase transition at around 280 °C [1]. This phase transition divides the thermal expansion behavior into two parts. Below the phase transition, the monoclinic low-temperature phase (LT-phase) is stable, which has a very high coefficient of thermal expansion (CTE) [2]. Above the phase transition, the orthorhombic high-temperature phase (HT-phase) is stabilized with its very low or even negative thermal expansion behavior [2]. The crystal structures of both phases are described in the literature. The LT-phase has the space group C2/c [1]. The space group of the HT-phase is Ccm2_1_ and is also reported in reference [1], where in-situ measurements were performed at high temperatures. However, the crystal structure was also refined in reference [3] using single crystals from a solid solution and a slightly different but very similar result (space group Cmcm) was obtained. However, the lattice parameters are defined differently in [1,3], which can easily lead to confusions. Hence, this work is based on the crystal structure reported in reference [3].

The BaZn_2_Si_2_O_7_ phase forms solid solutions within wide concentration ranges leading to a shift of the phase transition temperature depending on the site, which is occupied by other ions with the same valence state and similar ionic radii. A replacement of the Zn^2+^-ions by Mg^2+^, Mn^2+^, Co^2+^, Ni^2+^, and Cu^2+^ leads to a shift of the phase transition to higher temperatures, this is, a stabilization of the LT-phase in a wider temperature range [4]. If the Ba^2+^-ions are replaced by Sr^2+^, the phase transition temperature decreases and if a certain concentration of Sr^2+^ is exceeded, the HT-phase with its low thermal expansion is stable even below room temperature [3,4].

Materials containing high concentrations of alkaline earth oxides normally exhibit very high CTEs and a low thermal expansion might be unexpected or even undesired as in the case of sealing glasses or glass-ceramics for HT-reactors [5,6,7]. Hence, a detailed knowledge on the phase transition temperature for both the HT- as well as the LT-phase is necessary in order to control the thermal expansion behavior of materials, especially of glass-ceramics being able to precipitate the described solid solutions [8].

Furthermore, the crystalline solid solutions mentioned above exhibit CTE values, which strongly depend on the crystallographic direction and the composition. Especially in the case of phases with the structure of HT-BaZn_2_Si_2_O_7_, the CTEs of the different lattice parameters vary strongly [4,9]. The reason for this behavior is described in reference [3] to be caused by the crystal structure, which is composed of ZnO_4_ chains, running through the crystal in the direction of the lattice parameter c. These chains are connected by Si_2_O_7_ units. An increase of the temperature leads to a rotational movement of the ZnO_4_ tetrahedra and hence, the chains are stretched, which causes very high thermal expansion in the direction of the crystallographic c-axis. In the direction of the b-parameter, the ZnO_4_ tetrahedra are compressed, which causes highly negative thermal expansion.

This study reports on the influence of Ge^4+^ on the phase stability in Ba_0.5_Sr_0.5_Zn_2_Si_2-x_Ge_x_O_7_ and BaZnSi_2-x_Ge_x_O_7_ solid solutions prepared via solid-state reaction. Furthermore, the thermal expansion of the compound Ba_0.5_Sr_0.5_Zn_2_SiGeO_7_ in the different crystallographic directions was checked with high-temperature X-ray diffraction (HT-XRD).

## 2. Results and Discussion

**Figure 1:**
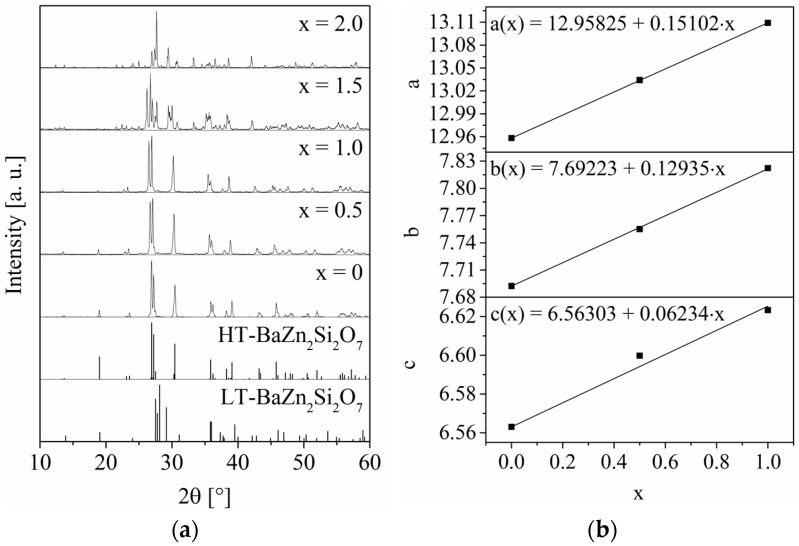
Results from X-ray diffraction (XRD) recorded at room temperature. (**a**) XRD patterns within the solid solution series Ba_0.5_Sr_0.5_Zn_2_Si_2-x_Ge_x_O_7_ with different values of x are shown. In the lower part, the theoretical peak positions of crystals with the structure of high-temperature (HT)- and low-temperature (LT)-BaZn_2_Si_2_O_7_ taken from references [3,11] are displayed; (**b**) the lattice parameters of Ba_0.5_Sr_0.5_Zn_2_Si_2-x_Ge_x_O_7_ are shown as a function of x. The values for x = 0 were taken from reference [9].

Figure 1 shows solid solutions of the form Ba_0.5_Sr_0.5_Zn_2_Si_2-x_Ge_x_O_7_ with different values of x. It can clearly be seen that samples with small Ge-concentrations exhibit the crystal structure of HT-BaZn_2_Si_2_O_7_ (see left part of Figure 1). The substitution of Si by Ge also leads to a shift of the peaks in the direction of smaller 2θ-values, i.e., larger lattice parameters, which is due to the larger ionic radius of Ge^4+^ in comparison to Si^4+^ [10]. The increasing lattice parameters are displayed at the right side of Figure 1 as a function of x together with the respective linear regression. The composition Ba_0.5_Sr_0.5_Zn_2_SiGeO_7_ still exhibits the crystal structure of HT-BaZn_2_Si_2_O_7_ without any impurity phases. At higher Ge-concentrations, the crystal structure of LT-BaZn_2_Si_2_O_7_ becomes stabilized together with some secondary phases, which cannot reliably be identified.

A similar behavior was found in the case of the solid solution without Sr. By contrast, these compositions exhibit the crystal structure of LT-BaZn_2_Si_2_O_7_ (diffractograms not illustrated here). The compound BaZn_2_SiGeO_7_ shows solely lines, which can be attributed to crystals with the structure of LT-BaZn_2_Si_2_O_7_. The compound BaZn_2_Si_0.5_Ge_1.5_O_7_ as well as the pure Ge-compound also show the crystal structure of LT-BaZn_2_Si_2_O_7_, but also some minor phases appear, which is in agreement with the findings reported in reference [12].

**Figure 2:**
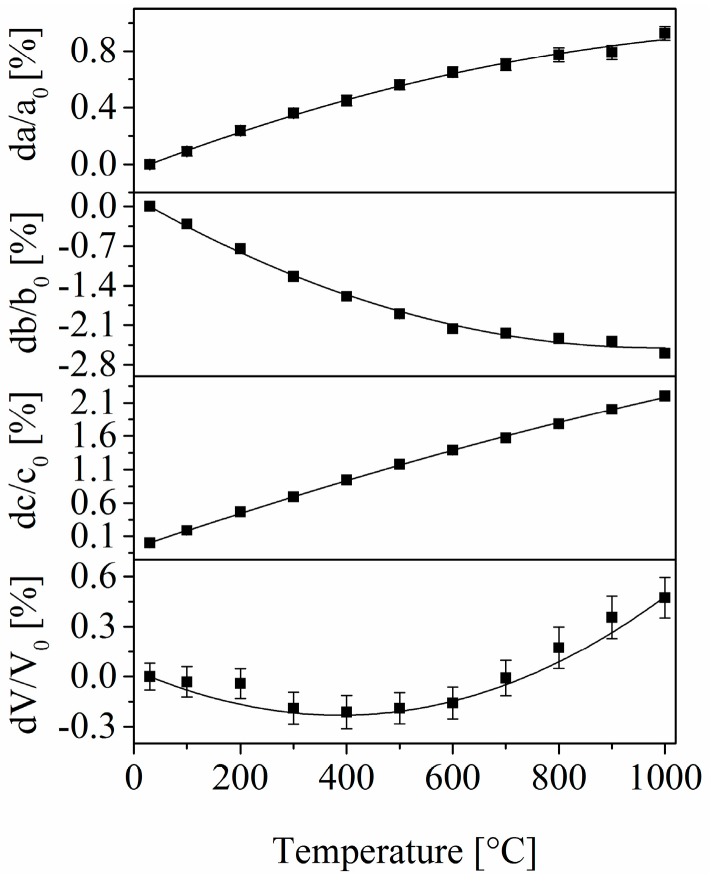
Relative change of the lattice parameters of Ba_0.5_Sr_0.5_Zn_2_SiGeO_7_ determined with HT-XRD. The values a_0_, b_0_, c_0_, and V_0_ were determined at room temperature. The line, which fits the change of the volume of the unit cell was calculated on the basis of the regression parameters given in Table 1.

The compound Ba_0.5_Sr_0.5_Zn_2_SiGeO_7_ was chosen in order to measure the thermal expansion behavior with HT-XRD. The lattice parameters of this composition can be fitted by second order polynomials. The corresponding regression parameters are summarized in Table 1. The relative change of the length of the lattice parameters a, b, and c as well as the volume of the unit cell V can be seen in Figure 2. As recently reported for the compound Sr_0.5_Ba_0.5_Zn_2_Si_2_O_7_ with this crystal structure, the lattice parameter b contracts strongly upon warming, whereas the a and the c parameters show an increasing length. The overall volume of the unit cell decreases with increasing temperature up to around 400 °C–500 °C. At higher temperatures, the volume of the unit cell increases. Between 600 and 1000 °C, this increase is almost linear.

The compound is highly anisotropic with CTEs of 13.4 × 10^−6^ K^−1^ (lattice parameter a), −45.7 × 10^−6^ K^−1^ (lattice parameter b), and 25.7 × 10^−6^ K^−1^ (lattice parameter c) measured between 30 and 300 °C. Between 30 and 800 °C, the anisotropy is a little bit smaller with CTEs of 10.1 × 10^−6^ K^−1^ (lattice parameter a), −30.4 × 10^−6^ K^−1^ (lattice parameter b), and 23.1 × 10^−6^ K^−1^ (lattice parameter c). The mean values of the respective CTEs are −2.2 × 10^−6^ K^−1^ (30 °C–300 °C) and 0.9 × 10^−6^ K^−1^ (30 °C–800 °C). These values are below those of the Ge-free compound Ba_0.5_Sr_0.5_Zn_2_Si_2_O_7_ and also below the values of most compositions where Zn^2+^ is replaced by other divalent transition metal ions or Mg^2+^ exhibiting the same crystal structure [9].

**Figure 3:**
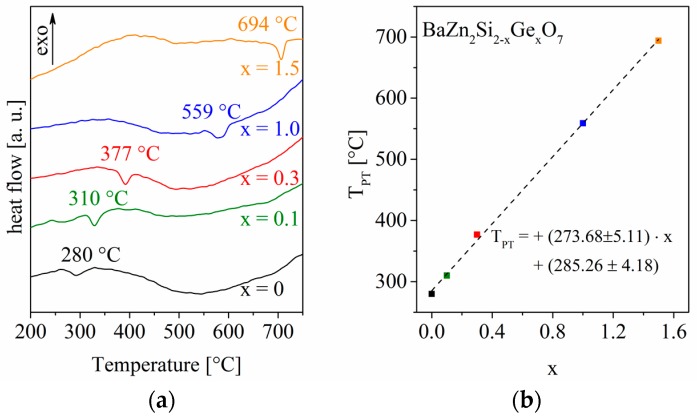
Phase transition temperatures T_PT_ of BaZn_2_Si_2-x_Ge_x_O_7_ solid solutions with different values of x. (**a**) the DSC curves are illustrated together with the respective onset temperatures of the phase transition; (**b**) the phase transition temperatures are plotted in dependence of x. The linear regression (dashed line and formula) is also inserted.

This should make such materials extremely resistant to thermal shock. However, obtaining a densely sintered material from such a highly anisotropic phase needs special techniques, such as sol-gel synthesis, in order to get crack-free materials [13]. If the Ge-concentration gets too high, the LT-modification becomes stabilized. In analogy, this can also be seen in the case of BaZn_2_Si_2-x_Ge_x_O_7_ solid solutions, exhibiting generally the crystal structure of LT-BaZn_2_Si_2_O_7_. These solid solutions show phase transitions to the HT-phase as illustrated in Figure 3. There it can be seen that an increasing Ge-concentration leads to an enlargement of the phase stability region of LT-BaZn_2_Si_2_O_7_, i.e., a shifting of the phase transition to higher temperatures, which is also observed for compounds in which the Zn^2+^-sites are substituted [2]. Further studies will be focused on the crystallization of phases with negative thermal expansion in order to achieve zero thermal expansion at room temperature and elevated temperatures.

## 3. Materials and Methods

Solid solutions within the series Ba_0.5_Sr_0.5_Zn_2_Si_2-x_Ge_x_O_7_ and BaZn_2_Si_2-x_Ge_x_O_7_ were prepared with different values of x from stoichiometric mixtures of SiO_2_ (>99%, Carl Roth GmbH & Co. KG, Karlsruhe, Germany), ZnO (≥99%, Carl Roth GmbH & Co. KG), BaCO_3_ (pure, VK Labor- und Feinchemikalien, Dresden, Germany), SrCO_3_ (purest, Ferak, Berlin, Germany), and GeO_2_ (>99.98%, ABCR GmbH & Co. KG, Karlsruhe, Germany). The respective powders were thoroughly mixed and afterwards heat treated at temperatures in the range from 1100 to 1200 °C kept for 20–30 h with several intermediate regrinding steps. Phase transition temperatures of the final powders were determined with differential scanning calorimetry DSC (LINSEIS DSC PT-1600, Selb, Germany). The phase analysis was performed with a SIEMENS D5000 Bragg-Brentano diffractometer (München, Germany) and Cu Kα radiation. The thermal expansion of Ba_0.5_Sr_0.5_Zn_2_SiGeO_7_ was determined up to 1000 °C with the same device equipped with an ANTON PAAR HTK 10 heating stage (Graz, Austria). For this purpose, the powdered samples were mixed with corundum in order to correct the height changes caused by the sample holder. Afterwards, the sample holder was heated with 5 K/s to the respective temperature. After a temperature equilibrium was reached, the scan was performed in the 2θ-range from 10° to 60° using an increment of Δ2θ = 0.02°. From the positions of the peaks, the lattice parameters were calculated with the software TOPAS 3 from BRUKER (Billerica, MA, USA).

## 4. Conclusions

The substitution of Si by Ge in HT- and LT-BaZn_2_Si_2_O_7_ polymorphs generally leads to the stabilization or the enlargement of the phase stability region of the LT-phase. In the case of Ba_0.5_Sr_0.5_Zn_2_SiGeO_7_, the HT-phase is still stable and the introduction of Ge decreases the CTE so that overall negative thermal expansion was measured with HT-XRD. That means, in the family of Ba_1-x_Sr_x_Zn_2-y_M_y_Si_2_O_7_ also the Si position, and hence all cationic positions can be substituted and nevertheless, negative thermal expansion can be achieved.

## Figures and Tables

**Table 1:** Regression parameters describing the temperature dependence of the lattice parameters a, b, and c of the compound Ba_0.5_Sr_0.5_Zn_2_SiGeO_7_ using a polynomial of the form: y(T) = A + BT + CT^2^.

Regression Parameters		a	b	c
**A (Å)**	**value**	13.10577	7.83397	6.61951
	**std. error.**	0.00266	0.00435	9.32275 × 10^−4^
**B (Å/°C)**	**value**	1.87277 × 10^−4^	−4.29385 × 10^−4^	1.77221 × 10^−4^
	**std. error.**	1.23447 × 10^−5^	2.0211 × 10^−5^	4.32873 × 10^−6^
**C (Å/°C^2^)**	**value**	−6.65004 × 10^−8^	2.19955 × 10^−7^	−2.78727 × 10^−8^
	**std. error.**	1.17341 × 10^−8^	1.92113 × 10^−8^	4.11461 × 10^−9^
